# Regional industrial growth and biopharma patent networks: empirical insights from the UK

**DOI:** 10.1007/s41109-022-00518-3

**Published:** 2022-11-16

**Authors:** Yuan Gao, Zhen Zhu

**Affiliations:** 1 School of Economics, University of East Anglia, Norwich, NR4 7TJ UK; 2 Department of Analytics, Operations and Systems, Kent Business School, University of Kent, Kent, CT2 7NZ UK

**Keywords:** Biopharmaceutical, Medical technology, Patent networks, Cross-border, Clustering comparison, Adjusted mutual information

## Abstract

The COVID-19 pandemic has once again brought the significance of biopharmaceutical and medical technology sectors to the spotlight. Seeing that some of the most critical medical breakthroughs such as the speedy mRNA vaccine development were results of cross-border patenting collaboration, we have proposed in a previous work a new method to identify the cross-border collaborative regional centres in the patent networks, using a clustering comparison approach based on adjusted mutual information (AMI). In this paper, we focus on the UK industrial landscape. We use the UK bioscience and health technology sector statistics from 2015 to 2020 and look into the regional growth of each postcode area. We compare the top growth regions with the cross-border collaborative centres identified using AMI comparison at the postcode area level, and find that both long-term and short-term AMI gains show an increase in the correlation with regional annual growth rates of firm numbers in the studied sectors from 2016 to 2020, and the increase is more consistent with the short-term AMI gain. We also found that areas more central in the long-term cross-regional R&D collaboration demonstrate a stronger association with more developed industrial settings indicated by more firms and, potentially more employment and turnover in the field. However, AMI gains are found to have negative correlations with the industrial growths as a sign of possible trade-offs of being central.

## Introduction

Globalisation and knowledge-based economy have stimulated the process of knowledge diffusion in the form of research and development (R&D) collaboration. Knowledge spillovers have been found to be geographically localised (Jaffe et al. 1993) and easier within firms than between (Kogut and Zander 1992). R&D collaboration between organisations in different countries could expose the participating parties to more heterogeneous resources, knowledge and skill sets. Data from the European Regional Innovation Survey from 1995 to 1997 has shown that manufacturing firms with an intensive innovation network are more successful, especially for the very small firms with stronger intraregional linkages (Koschatzky and Sternberg 2000). Conducting research on cross-border knowledge diffusion is especially meaningful as R&D cooperation and dissemination of innovation have been identified as key indicators in national innovation system (NIS) studies (Chessa et al. 2013; OECD et al. 1999; Chang and Shih 2004). In this paper, we focus on identifying regional centres in the cross-border collaborative networks as such centrality is associated with higher level of innovation intensity and quality. Our proposed identification method is based on the adjusted mutual information (AMI) gain by comparing each pair of elective partitions.

However, being a regional centre in a network can have restrictive effects on its productivity or performance in some circumstances. Such examples have been well documented and discussed in the network literature. Bettencourt et al. (2007) and Lobo and Strumsky (2008) have found that local interactions alone can lead to lock-in situations derived from the recirculation of homogeneous and redundant knowledge. Researchers have commented on global pipelines as a fundamental element in dealing with such issues because this allows to introduce external new knowledge that can be vital in the local innovation processes (Gertler 1995; Owen-Smith and Powell 2004).

In quantitative innovation studies, patent information has been a widely used data source in a series of important works (Griliches et al. 1986; Fleming 2001; Jaffe and Trajtenberg 2002; Hall et al. 2005). In the literature of R&D collaboration, researchers have been building linkages based on patent co-invention and co-application. In particular, the location information of patent inventors and applicants allows for accurate studies on cross-regional co-inventionship and talent mobility.

Maraut et al. coonstructed five networks using the OECD REGPAT database (Maraut et al. 2008) to explore the R&D integration in the European Union. These include the patent co-inventor and publication co-author networks, the patent co-applicant network, the patent citation network and the patent inventor mobility network. Singh’s analysis of patents filed to the U.S. Patent and Trademark Office (USPTO) uses patent citation data to measure the knowledge flow and builds interpersonal networks between inventors. In line with the previous literature like Kogut and Zander (1992), this analysis shows intra-regional and intra-firm knowledge flows are stronger than those across regional or firm boundaries (Singh 2005). On the temporal dimension, a study based on patents originated from OECD countries and filed through the European Patent Office (EPO) found that negative impact of geographical distance and institutional borders on R&D collaboration decreased from the end of 1980s till mid-1990s before it started to grow (Morescalchi et al. 2015). Further analysis looks into the impact of the quality of inter-regional knowledge networks constructed with the REGPAT patent database upon the regional research productivity (Sebestyén and Varga 2013). REGPAT is also used in combination with the Eurostat database with a focus on the innovation-lagging-behind European regions to suggest that having wider inter-regional co-patenting networks with closer collaboration with knowledge-intensive regions could help the less innovative regions to close the gap (De Noni et al. 2018).

As we have seen in the aforementioned literature, a rising number of researchers have come to recognise the importance of knowledge spillover. The earlier works look into various knowledge transmission channels (e.g., citation, collaboration, inventor mobility, etc.), and the more recent studies began to leverage the power of network methods. But still, a relatively smaller body of literature have come up with a method to measure the regional R&D network centrality. So far the most common approaches derive from the conventional social network analysis (SNA), such as degree centrality or betweenness centrality (Wanzenboeck et al. 2014; Wanzenböck et al. 2015). Berge et. al. argued that such studies could miss the conceptual problems at the aggregated level of regions and lose the information regarding the structure of network relations (Bergé et al. 2017). They proposed a new method based on the concept of inter-regional bridging paths defined as the indirect connections between two regions via a third region as the bridge.

When we go through the literature on cross-regional R&D collaboration and industry growth at a regional level, the NUTS3 level regions are commonly used as an international classification (Boschma and Iammarino 2009; Frenken et al. 2007; Van Stel and Nieuwenhuijsen 2004). However, the NUTS3 system cannot be well mapped to the LAU (Local Administrative Units) or the postal addresses. As most industry study resources available with UK firms come with their addresses, which primarily relies on the UK postcode, location identification using the postal data is more efficient.

Our analysis conducts network construction based on the cross-regional co-applicant linkages as they represent innovation collaboration between institutions. In terms of network centres identification, we take a different approach from the existing literature and use clustering comparison measures. Such measures have been traditionally used for external validation as well as clustering solutions search (Vinh et al. 2010). In this paper, we propose using clustering comparison in another application: as a way of identifying central nodes in networks. In the previous analysis (Zhu and Gao 2021), we have found that our proposed measure both correlates with and has advantages over the traditional measure of betweenness centrality as it better differentiates cross-border centres from local ones and offers a more uniform distribution of values. Our work also shows that compared to a simple measure of foreign share, AMI gain is more of a global and structural measure and better differentiates the nodes on the top.

In the rest of the paper we will introduce the data and method to measure regional industrial growth, followed by an introduction of the adapted AMI gain measure. Then we will present the results and conclude the paper with further discussions.

## Data and methods

### UK Bioscience and health technology sector statistics

The industrial data for this study is from the official collection of annual data on the bioscience and health technology sector in the United Kingdom (Office for Life Sciences 2021), published on the UK Government website. The statistics includes data on active firms based in the UK in the life science industry, by sector, segment, type of business activities, turnover band and employee band, as well as their address and website information. The collection starts from 2011, but varies in the type of data collected from year to year. We use the detailed data on firm level which is only available from 2015 to 2020, and extract the information with consistent definition and available in most of the years within this period.^1^ Table 1 lists out the key parameters from the raw data and the brief definition.^2^

**Table 1:** Key parameters extracted from UK Bioscience and Health Technology Statistics raw data

Type of data	Parameter name	Definition
Location	PACode	UK postcode area code
	region	NUTS region based on the postcode
Number of firms	Ntotal_2015-2020	annual number of all the biopharma and medical technology firms
- by sector	NBPCore_2015-2020	Biopharmaceutical Core sector
	NBPSvc_2015-2020	Biopharmaceutical Service and Supply Chain sector
	NMTCore_2015-2020	Medical Technology Core sector
	NMTSvc_2015-2020	Medical Technology Service and Supply Chain sector
- by turnover band	Nt1_2015, 2017-2020	0-£49K
	Nt2_2015, 2017-2020	£50K-£99K
	Nt3_2015, 2017-2020	£100K-£249K
	Nt4_2015, 2017-2020	£250K-£499K
	Nt5_2015, 2017-2020	£500K-£999K
	Nt6_2015, 2017-2020	£1M-£5M
	Nt7_2015, 2017-2020	£5M+
- by employment band	Ne1_2015-2020	0-4
	Ne2_2015-2020	5-9
	Ne3_2015-2020	10–19
	Ne4_2015-2020	20–49
	Ne5_2015-2020	50–99
	Ne6_2015-2020	100–249
	Ne7_2015-2020	250+

PACode is the first one or two alphabetic digits before the first numeric digit in a UK postcode, indicating the postal area for the UK Royal Mail delivering purpose. There are 125 postcode areas in total. We have extracted the PACodes from the firm addresses and mapped them to area names by referring to the ONS Postcode Directory (February, 2022) (Office for National Statistics 2022). Among the four sectors, the two “Cores” (Biopharmaceutical Core and Medical Technology Core) include businesses involved in developing and/or producing pharmaceutical or medical technological products, and the other two (Biopharmaceutical Service and Supply Chain and Medical Technology Service and Supply Chain) comprise businesses offering goods and services to the Core businesses (Office for Life Sciences 2022). The raw data doesn’t include the exact figures of firms’ turnover or employment, but reports them in bands.

Based on the extracted raw data, we calculate the year-to-year industrial growth of each postcode area in terms of the number of firms registered there, simply put as Eq. 1, where $G_{tp}$ represents the growth of postcode area *p* in year *t*, $N_{tp}$ the number of postcode area *p* in year *t*, and $N_{(t-1)p}$ the number of postcode area *p* in year *t* − 1. Although not specified in the equation, $N_{tp}$ and $G_{tp}$ shall be interpreted as general terms as the number and growth of firms per sector, revenue band or employee band as applicable.

1 $$\begin{aligned} G_{tp} = \frac{N_{tp} - N_{(t-1)p}}{N_{(t-1)p}} \end{aligned}$$

**Table 2:** Variables and definitions: Annual irm numbers

Variable name	Definition
Ntotal	Annual number of firms: both biopharma and medical technology field
NBPCore	Annual number of firms: Biopharmaceutical Core sector
NMTCore	Annual number of firms: Medical Technology Core sector
NBPSvc	Annual number of firms: Biopharmaceutical Service and Supply Chain sector
NMTSvc	Annual number of firms: Medical Technology Service and Supply Chain sector
Nt1	Annual number of firms: turnover band 0-£49K
Nt2	Annual number of firms: turnover band £50K-£99K
Nt3	Annual number of firms: turnover band £100K-£249K
Nt4	Annual number of firms: turnover band £250K-£499K
Nt5	Annual number of firms: turnover band £500K-£999K
Nt6	Annual number of firms: turnover band £1M-£5M
Nt7	Annual number of firms: turnover band £5M+
Ne1	Annual number of firms: employment band 0-4
Ne2	Annual number of firms: employment band 5-9
Ne3	Annual number of firms: employment band 10-19
Ne4	Annual number of firms: employment band 20-49
Ne5	Annual number of firms: employment band 50-99
Ne6	Annual number of firms: employment band 100-249
Ne7	Annual number of firms: employment band 250+

Tables 2 and 3 list the variable names and definitions. We also calculate the average annual numbers of firms and average annual growths, generally denoted as $avg\_N_{p}$ and $avg\_G_{p}$

**Table 3:** Variables and definitions: Annual firm number growth rates

Variable name	Definition
Gtotal	Annual growth based on firm numbers: both biopharma and medical technology field
GBPCore	Annual growth based on firm numbers: Biopharmaceutical Core sector
GMTCore	Annual growth based on firm numbers: Medical Technology Core sector
GBPSvc	Annual growth based on firm numbers: Biopharmaceutical Service and Supply Chain sector
GMTSvc	Annual growth based on firm numbers: Medical Technology Service and Supply Chain sector
Gt1	Annual growth based on firm numbers: turnover band 0-£49K
Gt2	Annual growth based on firm numbers: turnover band £50K-£99K
Gt3	Annual growth based on firm numbers: turnover band £100K-£249K
Gt4	Annual growth based on firm numbers: turnover band £250K-£499K
Gt5	Annual growth based on firm numbers: turnover band £500K-£999K
Gt6	Annual growth based on firm numbers: turnover band £1M-£5M
Gt7	Annual growth based on firm numbers: turnover band £5M+
Ge1	Annual growth based on firm numbers: employment band 0-4
Ge2	Annual growth based on firm numbers: employment band 5-9
Ge3	Annual growth based on firm numbers: employment band 10-19
Ge4	Annual growth based on firm numbers: employment band 20-49
Ge5	Annual growth based on firm numbers: employment band 50-99
Ge6	Annual growth based on firm numbers: employment band 100-249
Ge7	Annual growth based on firm numbers: employment band 250+

### AMI gain algorithm

The development of the AMI gain method is detailed in our previous work (Zhu and Gao 2021). We provide a brief review here: For the constructed network with weighted links, we restrict our focus to the largest components and use the Louvain method (Blondel et al. 2008) for community detection. In the detected network partition, we apply clustering comparison method by measuring and comparing the similarity scores of a clustering before and after arbitrarily removing cross-border links of a focal node against the default clustering defined by national administrative borders. The difference between the similarity scores is the AMI gain of the focal node. In other words, the more adjusted mutual information the network could gain by having a node, the more central the node is.

The original AMI methodology has been adapted for this study: First, the original method is based on NUTS3 level region division, while in this paper it’s been revised to map to the UK postcode areas. Second, in this study we combine pharmaceuticals and biotechnology patents together. And third, instead of using all the patents with priority dates from 1976, we now focus on two periods: 1976–2014 and 2010–2014, representing effects of the long-term accumulation of cross-regional innovation cooperation heritage and the short-term one, respectively, on the observed period of industry growth. Same as the previous work, the patent data we use is still the OECD REGPAT database (released in January, 2021) (Maraut et al. 2008).

We now explain the adaption in details. This analysis focuses on the 30 countries in Europe, i.e., the EU28 countries except for Cyprus before the Brexit, plus Iceland, Norway and Switzerland. For the United Kingdom, we use postcode areas of the patent applicant addresses as the network nodes. For the other countries, we still follow the NUTS3 level regions. The cross-border links between the UK and the other European countries are, therefore, between any UK postcode areas and another country’s NUTS3 regions. For each UK postal area, the links with other UK postal areas and with other European regions are equally considered. Patents categorised into both the biotechnology and pharmaceuticals fields according to the IPC concordance table published by the WIPO (WIPO 2019) are used in the dataset to construct a combined bio-pharmaceutical co-applicant network. The links are weighted by the accumulated number of co-applicant collaboration instances between UK postal areas and NUTS3 regions over time (i.e., from 1976 to 2014, or from 2010 to 2014). As in the previous study, self-loops are considered and weighted.

We denote the network as $G=(V,E)$ where *V* is the set of nodes (or vertices) and *E* is the set of links (or edges). To highlight the changes in this study, we further denote $V=V_1$ $\cup$ $V_2$, where $V_1$ as the set of nodes of UK PACodes, and $V_2$ as the set of nodes of the NUTS3 regions in other countries. Despite the different definition of regional division, nodes from both subsets are treated the same in network edge construction and partitioning.

The definiton of AMI in mathmatical formula is the same as in the previous work (Zhu and Gao 2021). Algorithm 1 shows the adapted pseudocode of calculating the AMI gain for each node. $v_i$ $\in$ *V* represents node *i* in the network, and $e_{v_i,v_j}$ $\in$ *E* as the edge between node *i* and node *j*. The set of node *i*’s neighbouring (directly connected) nodes is denoted as $N(v_i)$. The largest component of the network is denoted as $C_1$. A cluster containing node *i* is denoted as $P_i$, and the cluster after node *i* has been removed is denoted as $P_{-v_i}$.
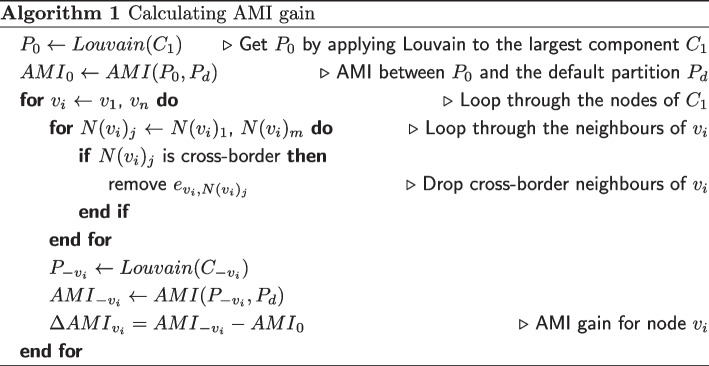


## Results

We now focus on the correlation between the long-term and short-term patent co-applicant network AMI gains (denoted as $amigain\_1976$ and $amigain\_2010$, respectively) and the regional industry status, i.e. the number of firms and their growths in each UK postcode area. It is noteworthy that although the firms have covered 122 out of all the 125 PACodes, not all of the areas have patent-producing firms. In fact, from 1976 to 2014, 88 postal areas have actually generated bio-pharmaceutical patents, and from 2010 to 2014 only 54.

**Fig. 1:**
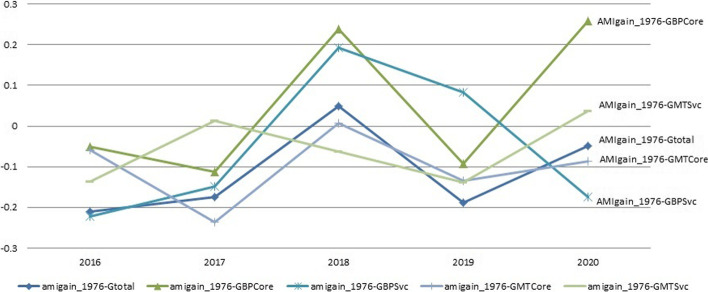
Correlations between AMI Gain (from 1976 to 2014) and Annual Firm Numbers and Growths by Sector

**Fig. 2:**
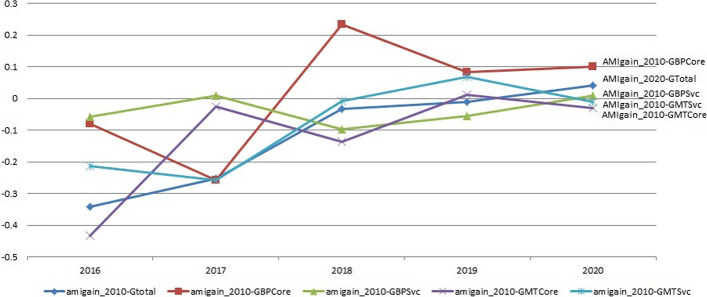
Correlations between AMI gain (from 2010 to 2014) and annual firm numbers and growths by sector

We first examine the correlations between AMI gains over the longer and shorter periods and the regional firm quantity growth rates in each year, shown in Fig. 1 (AMI gain from 1976 to 2014) and Figure 2 (AMI gain from 2010 to 2014). Figure 1 shows that 2018 marks a year with overall high correlations followed by a drop in the next year. All the growth indicators but the one in biopharmaceutical service and supply chain sector pick up in 2020. All the correlations in 2020 are higher than 2016. This uprising trend is more consistent and stronger in Fig. 2. These two figures show that the correlations with both long-term and short-term AMI gains have been increasing in the recent years, and the increase is more stable with the short-term AMI gain.

Table 2 shows the pairwise correlation coefficients between AMI gains and the average firm numbers and growths. It is noticeable that the correlations between the long-term AMI gain and the number of firms are mostly positive, while the short-term AMI gain shows more negative correlations. This indicates that a longer-term heritage of cross-regional R&D collaboration is associated with higher firm numbers. Such positive correlations are more significant with separate sectors (Medical Technology Core and Medical Technology Service), turnover bands (0-£49K,£500K-£999K, £1M-£5M), and employment band (5–9).

Negative correlation coefficients are observed between AMI gains and most growth measures. Indeed, as mentioned in the literature introduction, centrality could have possible negative effects on node performance. Bianchi et al. (2021) analysed the trade-offs of brokerage at a similar scale as ours and found that cities holding a central position in the inter-city innovation collaboration networks show higher patenting activity level, while being a broker can negatively influence patenting outcomes. Another element for consideration is that an area with a well-established industry can naturally present relatively lower growth rates for a given number of new firms. More specifically, a more established area over the last 40 years would need to have more newly registered firms to achieve the same level of growth of a less established and emerging area.

Here we document the findings without determining any causal links. As the patent data used to calculate AMI gains ends at 2014, the rising trend and changing signs of the correlations shown in Figure 1 and 2 could indicate that there is a time lag of 2–3 years between cross-regional R&D collaboration and its influence on the regional industry.

Furthermore, we would like to highlight the stronger correlations between long-term AMI gain and the medical technology firm numbers. According to the industry statistics in 2020 (Office for Life Sciences 2022), the Med Tech Core sector is the largest in the industry by employment (106,500 total employees, 40% of the industry) and number of firms (2,900 in total, 46% of the industry). Its supporting Service and Supply Chain sector also contributes significant shares: 63,900 employees and 1,690 businesses. In fact, approximately 138,100 (52% of the industry total) are employed in the Med Tech sectors. This could suggest that cross-regional patenting efforts can be associated with boosting the regional entrepreneurship and employment. The significant positive correlation between long-term AMI gain and number of firms in turnover band 5 and 6 also suggests that an accumulated cross-regional innovation heritage can be linked with increasing the number of highly profiting firms in an area.

**Fig. 3:**
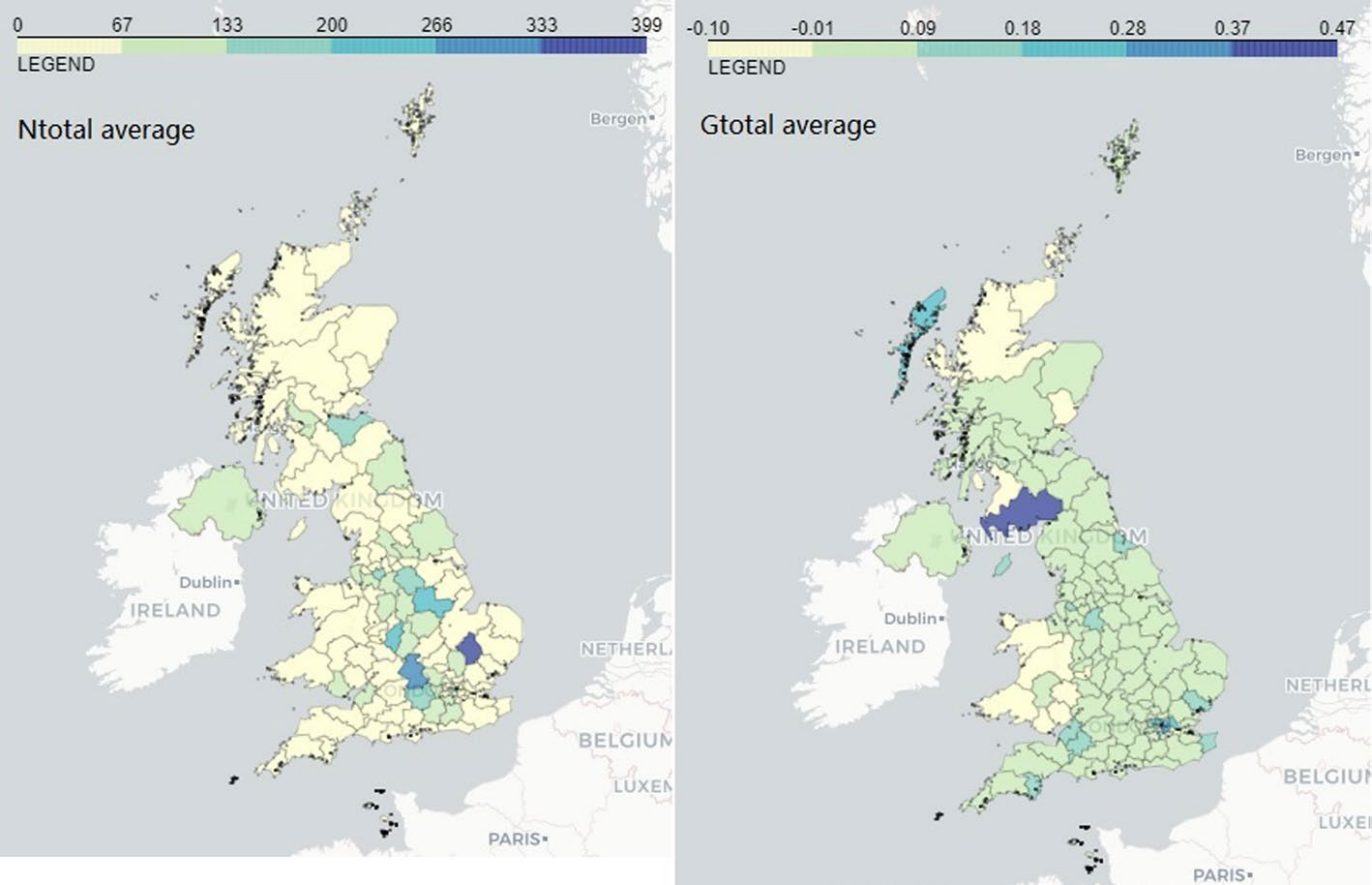
UK postcode areas with numbers and growths of firms in the bioscience and health technology industry

In Fig. 3, the left panel shows the average number of firms in the entire bioscience and health technology industry from 2015 to 2020 of each postcode area plotted on the UK map. A region marked with deeper color indicates higher number of firms. The right panel shows the average growth from 2016 to 2020 in the similar fashion. The illustration shows that areas with the most firms do not necessarily overlap with the fast-growing areas. For example, Comhairle nan Eilean Siar and Dumfries. Cambridge is an advanced area with a large number of firms in the field (399), and its surrounding areas show the similar level of growth as it.

**Fig. 4:**
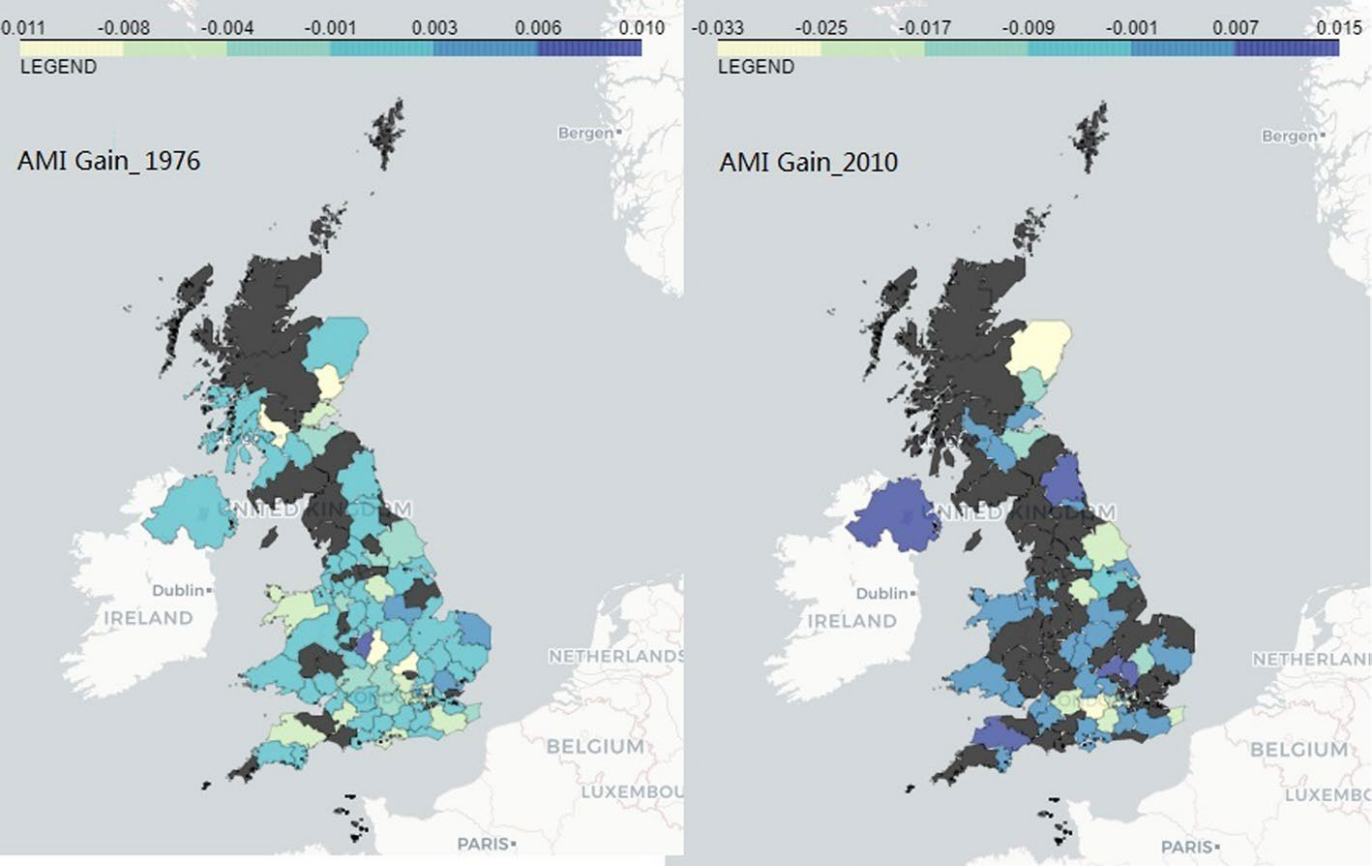
UK postcode areas with AMI gains in the pharmaceuticals and biotechnology sectors

Figure 4 shows the long-term (left panel) and short-term (right panel) regional AMI gain on the UK map, in which the black areas are the non-patent-producing regions. As the color goes keeper, the AMI gain increases. We can see that the short-term AMI gain is at a higher level compared to the long-term, with some more outstanding areas like Belfast, Newcastle, Glasgow, Dundee, Milton Keynes, Stevenage and Exeter, other areas apparently engaging in less cross-regional patenting activities like Aberdeen, and some areas not having produced any patents during the more recent period of 2010–2014 (most of them have a small number of patents even since 1976). Comparing Fig. 3 and 4, the long-term areas more active in cross-regional invention collaboration overlap with the areas with more firms and higher growths mainly in the regions of East Midlands, East of England, London and South East (Table 4).

**Table 4:** Pairwise correlation between AMI gains and the average number of firms and the average growths

Average numbers	amigain_1976	amigain_2010	Average Growths	amigain_1976	amigain_2010
avg_Ntotal	0.1559	−0.0439	avg_Gtotal	−0.0857	−0.2189
	88	54		88	54
avg_NBPCore	0.0579	−0.2621*	avg_GBPCore	0.0616	−0.0951
	81	53		81	53
avg_NMTCore	0.1838*	0.0807	avg_GMTCore	−0.0915	−0.2790*
	88	54		88	54
avg_NBPSvc	0.0804	−0.1039	avg_GBPSvc	−0.0012	−0.0753
	87	54		87	54
avg_NMTSvc	0.2453*	0.0555	avg_GMTSvc	−0.0588	−0.1979
	87	54		87	54
avg_Nt1	0.2002*	−0.0221	avg_Gt1	−0.0649	−0.0716
	88	54		88	54
avg_Nt2	0.1341	−0.0580	avg_Gt2	−0.0384	0.1431
	86	54		86	54
avg_Nt3	0.1545	−0.0422	avg_Gt3	0.0757	0.0307
	86	54		86	54
avg_Nt4	0.1590	−0.0684	avg_Gt4	−0.1662	0.0399
	87	54		87	54
avg_Nt5	0.1795*	0.0713	avg_Gt5	−0.1063	−0.1869
	87	54		87	54
avg_Nt6	0.1836*	−0.0127	avg_Gt6	0.0193	0.0215
	87	54		87	54
avg_Nt7	0.0611	−0.1220	avg_Gt7	0.0500	−0.1919
	88	54		88	54
avg_Ne1	0.1602	−0.0690	avg_Ge1	−0.0011	−0.0631
	88	54		88	54
avg_Ne2	0.2173*	−0.0205	avg_Ge2	−0.2739*	−0.3216*
	87	54		87	54
avg_Ne3	0.1723	0.0807	avg_Ge3	−0.1780*	−0.1761
	87	54		87	54
avg_Ne4	0.1159	−0.0614	avg_Ge4	−0.1567	−0.2451*
	86	54		86	54
avg_Ne5	0.1029	−0.1241	avg_Ge5	−0.0489	−0.2505*
	83	52		83	52
avg_Ne6	0.0764	−0.0381	avg_Ge6	−0.1560	0.0059
	83	52		83	52
avg_Ne7	0.0643	−0.1740	avg_Ge7	0.1187	0.1953
	78	52		78	52

*$p<0.05$. For each pairwise correlation, the first value is the correlation coefficient and the value below is the number of observations. The average growth by turnout band is based on the growth in year 2018–2020 only

## Conclusion and future work

R&D collaborations beyond national borders are critical for knowledge spillovers at large scale, which is well demonstrated by the recent development of COVID19 mRNA vaccines at an unprecedented timescale. This paper focuses on the UK’s bioscience and health technology industry and uses a new government-released industry dataset to propose a different perspective into the impact of cross-regional innovation on the industry.

This paper demonstrates a new application of the previously proposed clustering comparison approach based on adjusted mutual information. We associate the network method with real-world industry data, and therefore contribute to the literature by exploring the relationship with the regional industrial growths with the cross-region patent collaboration “centralness”. Regional analysis on the level of UK postcode areas is a relatively untapped field in the literature, and this study contributes to filling this gap.

We present two key findings through this study. One, an increase is observed in the correlations between both long-term and short-term AMI gains and annual growth rates of firm numbers in UK’s bioscience and health technology sectors from 2016 to 2020, and the increase is more consistent with the short-term AMI gain. Two, in terms of the correlation with average regional firm numbers from 2016 to 2020, the long-term AMI gain shows more positive and higher significance than the short-term AMI gain. In the meantime, we also observe and discuss the negative correlation between regional AMI gains and the industrial growth rates. This adds to the literature of potential trade-offs of centrality in a network.

Given that most of the nodes in the patent co-application network are foreign regions outside of the UK, it is likely that the links with overseas businesses play a bigger role in the AMI gain. The patent dataset ends by 2014, before the 2016 referendum on the UK’s EU membership, let alone the actual withdrawal process later. This paper has not differentiated the domestic collaborations from the foreign ties, which is worth exploring in future work to provide insights on the potential impact of Brexit on the biopharma and medical technology sectors in the UK.

The authors of this paper are not specialised in UK regional policies and initiatives in the relevant industry. There are, undoubtedly, other not insignificant factors in the regional industrial growth, such as public and private investments, entrepreneurship stimulus, talents movements, etc. Challenge of obtaining such data results in a major limitation of this study. We look forward to completing the work and exploring more of the trade-off effects if a more thorough dataset becomes available.

### Supplementary Information

**Additional file 1.** Appendix 1.csv.

## Data Availability

Patent data used in this study is publicly available from the OECD REGPAT database released in January, 2021 (Maraut et al. 2008). The industrial firms data used in this study is from the official collection of annual data on the bioscience and health technology sector in the United Kingdom from 2015 to 2020 (Office for Life Sciences 2021). Other relevant data and materials are available upon request.

## Appendix

Appendix 1: List of UK postcode areas and the name of areas and regions. See Additional file 1: Appendix 1.csv.

## Footnotes

1 Turnover band in 2016 was collected in an alternative metric other than the rest of the years. Therefore, it’s not included in this study.

2 We applied manual data harmonisation due to different formats and naming conventions of the raw data in each year.
